# The IFN-γ-related long non-coding RNA signature predicts prognosis and indicates immune microenvironment infiltration in uterine corpus endometrial carcinoma

**DOI:** 10.3389/fonc.2022.955979

**Published:** 2022-07-26

**Authors:** Chunyan Gu, Chen Lin, Zheng Zhu, Li Hu, Fengxu Wang, Xuehai Wang, Junpu Ruan, Xinyuan Zhao, Sen Huang

**Affiliations:** ^1^ Department of Obstetrics and Gynecology, Nantong Haimen People’s Hospital, Nantong, China; ^2^ Vectors and Parasitosis Control and Prevention Section, Center of Disease Prevention and Control in Pudong New Area of Shanghai, Shanghai, China; ^3^ Department of Urology, The First Affiliated Hospital of Nanjing Medical University, Nanjing, China; ^4^ Department of Medicine, Kangda College of Nanjing Medical University, Lianyungang, China; ^5^ Department of Occupational Medicine and Environmental Toxicology, Nantong Key Laboratory of Environmental Toxicology, School of Public Health, Nantong University, Nantong, China

**Keywords:** interferon-gamma, bioinformatics, signature, immunology, endometrial carcinoma

## Abstract

**Background:**

One of the most common diseases that have a negative impact on women’s health is endometrial carcinoma (EC). Advanced endometrial cancer has a dismal prognosis and lacks solid prognostic indicators. IFN-γ is a key cytokine in the inflammatory response, and it has also been suggested that it has a role in the tumor microenvironment. The significance of IFN-γ-related genes and long non-coding RNAs in endometrial cancer, however, is unknown.

**Methods:**

The Cancer Genome Atlas (TCGA) database was used to download RNA-seq data from endometrial cancer tissues and normal controls. Genes associated with IFN-γ were retrieved from the gene set enrichment analysis (GSEA) website. Co-expression analysis was performed to find lncRNAs linked to IFN-γ gene. The researchers employed weighted co-expression network analysis (WGCNA) to find lncRNAs that were strongly linked to survival. The prognostic signature was created using univariate Cox regression and least absolute shrinkage and selection operator (LASSO) regression. The training cohort, validation cohort, and entire cohort of endometrial cancer patients were then split into high-risk and low-risk categories. To investigate variations across different risk groups, we used survival analysis, enrichment analysis, and immune microenvironment analysis. The platform for analysis is R software (version X64 3.6.1).

**Results:**

Based on the transcript expression of IFN-γ-related lncRNAs, two distinct subgroups of EC from TCGA cohort were formed, each with different outcomes. Ten IFN-γ-related lncRNAs were used to build a predictive signature using Cox regression analysis and the LASSO regression, including CFAP58, LINC02014, UNQ6494, AC006369.1, NRAV, BMPR1B-DT, AC068134.2, AP002840.2, GS1-594A7.3, and OLMALINC. The high-risk group had a considerably worse outcome (*p* < 0.05). In the immunological microenvironment, there were also substantial disparities across different risk categories.

**Conclusion:**

Our findings give a reference for endometrial cancer prognostic type and immunological status assessment, as well as prospective molecular markers for the disease.

## Introduction

Endometrial carcinoma generally refers to cancer of the corpus uteri, which is a common malignant tumor of the female reproductive system. Since it has increasingly become the main cause of cancer death, early diagnosis is important. In 2020, the incidence rate of EC ranks sixth in the female population (1). In 2021, cancer statistics reported the estimation, which conjectures the fourth incidence rate and sixth mortality of EC in the United States (2). Citizens of developed countries have a high risk of developing EC owing to obesity and lack of exercise (3). EC could be categorized into type I and type II (3). The former type is usually related to excessive estrogen expression, while the latter is often estrogen-independent, including clear cell carcinoma and serous carcinoma (4). Segmental curettage and endometrial biopsy are efficient diagnostic approaches. Although patients with early-stage endometrial cancer have better results after surgical treatment, there are still some patients who are diagnosed at an advanced stage and lose the opportunity for surgery (5, 6). Moreover, the treatment of estrogen-independent EC and undifferentiated EC remains a challenge (7). The use of immune checkpoint inhibitors and angiogenesis inhibitors has yielded encouraging results in patients with advanced, hormone-independent, or undifferentiated EC (8). However, EC patients also have significant heterogeneity, with different tumor immune reprogramming states and microsatellite instability phenotypes, resulting in varying degrees of response to immunotherapy (9). Therefore, it is necessary to explore the tumor microenvironment of EC to provide a reference for treatment.

Interferon-γ (IFN-γ) is common in the tumor microenvironment and body inflammation (10). IFN-γ is produced primarily by T and NK cells in response to various inflammatory or immune stimuli (11). The role of IFN-γ in immune surveillance and immune escape of tumors has been demonstrated. Many studies have indicated an increase in IFN-γ production during immune checkpoint blocking therapy (12). Defects in IFN-γ signaling are associated with resistance to immunotherapy. Thus, IFN-γ plays a key role in the tumor microenvironment (13). On the one hand, it serves as an immunogenicity enhancer *via* upregulating the expression of MHC and genes required in antigen processing (14). On the other hand, IFN-γ could combine with PD-1 on tumor-infiltrating T cells, inhibiting tumor immune regulation (15). Apart from them, researchers had indicated that IFN-γ acts as a regulator of hematopoietic stem cells in both homeostasis and during infection (16). A meta-analysis by Deng et al. (17) also showed the significant relationship between IFN-γ polymorphisms +874 (T/A) and the occurrence risk of aplastic anemia. Additionally, IFN-γ alone could moderately suppress tumor cell growth by inducing apoptosis in, for example, ovarian cancer (18).

Non-coding RNA longer than 200 nt is known as long non-coding RNA (lncRNA), and it is now thought that lncRNA plays a significant role in the development and spread of cancer (19). There is growing evidence that lncRNA can play a role in the regulation of gene expression and transcription at the transcriptional and epigenetic levels, as well as the important regulatory processes of chromatin modification, transcription activation, and genomic imprinting (20). It also plays a role in the development of complex precision in gene expression, as well as tumor growth, apoptosis, invasion, metastasis, and many other biological processes (21). However, the role of IFN-γ-related lncRNAs in endometrial cancer has hardly been studied.

In this study, we explored the significance of IFN-γ-related genes in endometrial carcinoma and constructed prognostic signatures through comprehensive analysis. With this signature, the prognosis and immune status of patients with endometrial cancer can be well assessed and stratified, thus providing a reference for the diagnosis and treatment of endometrial cancer.

## Methods

### Data acquisition

The RNA-seq transcriptome data in fragment per kilobase method (FPKM) format and corresponding clinical data of uterine corpus EC (UCEC) patients were extracted from The Cancer Genome Atlas (TCGA) (UCEC tissue samples, 552; normal samples, 23). After clinical information was combined with transcriptome data, 511 tumor samples were obtained. The 511 tumor samples were evenly divided into the training cohort (256 samples) and validation cohort (255 samples), at a 1:1 ratio. Subsequently, these data were collated, annotated, and then collapsed into protein-coding genes and lncRNAs by employing the annotation documents from the GENCODE database. A total of 13,349 lncRNAs were identified (22). The IFN-γ-related genes were obtained from the HALLMARK_INTERFERON_GAMMA_RESPONSE genome of gene set enrichment analysis (GSEA). Subsequently, Pearson’s correlation analysis was conducted using the 13,349 lncRNAs and IFN-γ-related genes (*p* < 0.001, correlation coefficient > 0.3). Ultimately, 1,700 IFN-γ-related lncRNAs were screened for follow-up bioinformatics analysis. **Supplementary Table 1** shows the clinical data of UCEC patients obtained from TCGA. We used R software to extract the expression of IFN-γ-related lncRNAs for further investigation.

### Weighted gene co-expression network analysis

To develop a scale-free co-expression network in EC, genes related to IFN-γ based on the 25th percentile of variance were selected by using the weighted gene co-expression network analysis (‘WGCNA’) package (23). For all IFN-γ-related genes, during this time, Pearson’s correlation and average linkage algorithm were performed, and a weighted adjacency matrix was constructed (MMi = |cor(x(i)), ME|, where i is the value of each gene) (24). Then, average linkage modules were clustered, and the further dissimilarity of module IFN-γ-related genes was detected.

### Establishment and validation of the prediction model

Prognostic IFN-γ-related lncRNAs were screened out *via* univariate Cox regression analysis. Further, a prediction model based on IFN-γ-related lncRNAs expression was established through least absolute shrinkage and selection operator (LASSO) analysis, and the formula of the prediction model was as follows: Risk score = coef * Exp (lncRNA A) + coef * Exp (lncRNA B) + coefi * Expi (lncRNA i) (25).

EC patients were divided into two groups based on the training cohort’s median risk score. The group with a greater risk score than the median was labeled as high risk. The rest of them were in the low-risk category. The overall survival (OS) of the two groups was calculated using the Kaplan–Meier (KM) analysis (26), and the reliability of the prediction model was assessed using receiver operating characteristic (ROC) curve analysis (27). The innovative prediction model was verified using the same methodology in the test and entire TCGA cohorts.

### Evaluation of the prediction model

GSEA study was performed using the Java GSEA program with 1,000 random permutations to investigate the varied biological activities of the high- and low-risk groups based on IFN-γ-related lncRNAs (28). In accordance with past findings, immune cell infiltration affects the survival and tumor metastasis of patients. The immune cell infiltration of the two groups was investigated using two distinct techniques, the CIBERSORT algorithm (29) and the ssGSEA algorithm (30). The immune cell infiltration of EC patients in different groups was compared using the Wilcoxon test, with a *p*-value of 0.05 considered statistically significant. Furthermore, the expression of genes related to immunological checkpoints, *N* ^6^-methyladenosine RNA methylation, and the stem cell pathway was calculated in two groups, with a *p*-value of 0.05 considered statistically significant.

### Independent prognostic analysis and construction of a nomogram

Univariate and multivariate Cox regression analyses were conducted to determine if the prediction model we constructed could be used as an independent predictor of survival in EC patients. A nomogram was then constructed based on the independent prognostic factors by the ‘rms’ package in R software. Calibration curves and ROC curves were used to verify the validity of the prediction model.

### Quantitative real-time polymerase chain reaction

The 15 EC patients whose EC tissue and normal uterine tissue were collected for mRNA quantification were then subjected to qRT-PCR analysis. Following the manufacturer’s directions, total cellular RNAs were extracted from cells using Trizol Reagent (Invitrogen, Carlsbad, CA, USA). Reverse transcription was done with the Takara reverse transcription kit (Otsu, Shiga, Japan). Thermo Fisher Scientific (Waltham, MA, USA) provided the QuantiTect SYBR Green PCR Kit and QuantStudio 1 for the real-time polymerase chain reaction (RT-PCR). The −2^ΔΔCt^ technique was used to determine relative quantification. Each gene’s relative messenger RNA (mRNA) expression level was adjusted to that of the mRNA for the enzyme glyceraldehyde-3-phosphate dehydrogenase (GAPDH). The primer sequences used are shown in **Supplementary Table 2**.

### Statistical analysis

R software was used to conduct all statistical analyses (version x64 3.6.1). A *p*-value of less than 0.05 was considered statistically significant.

## Result

**Figure 1:**
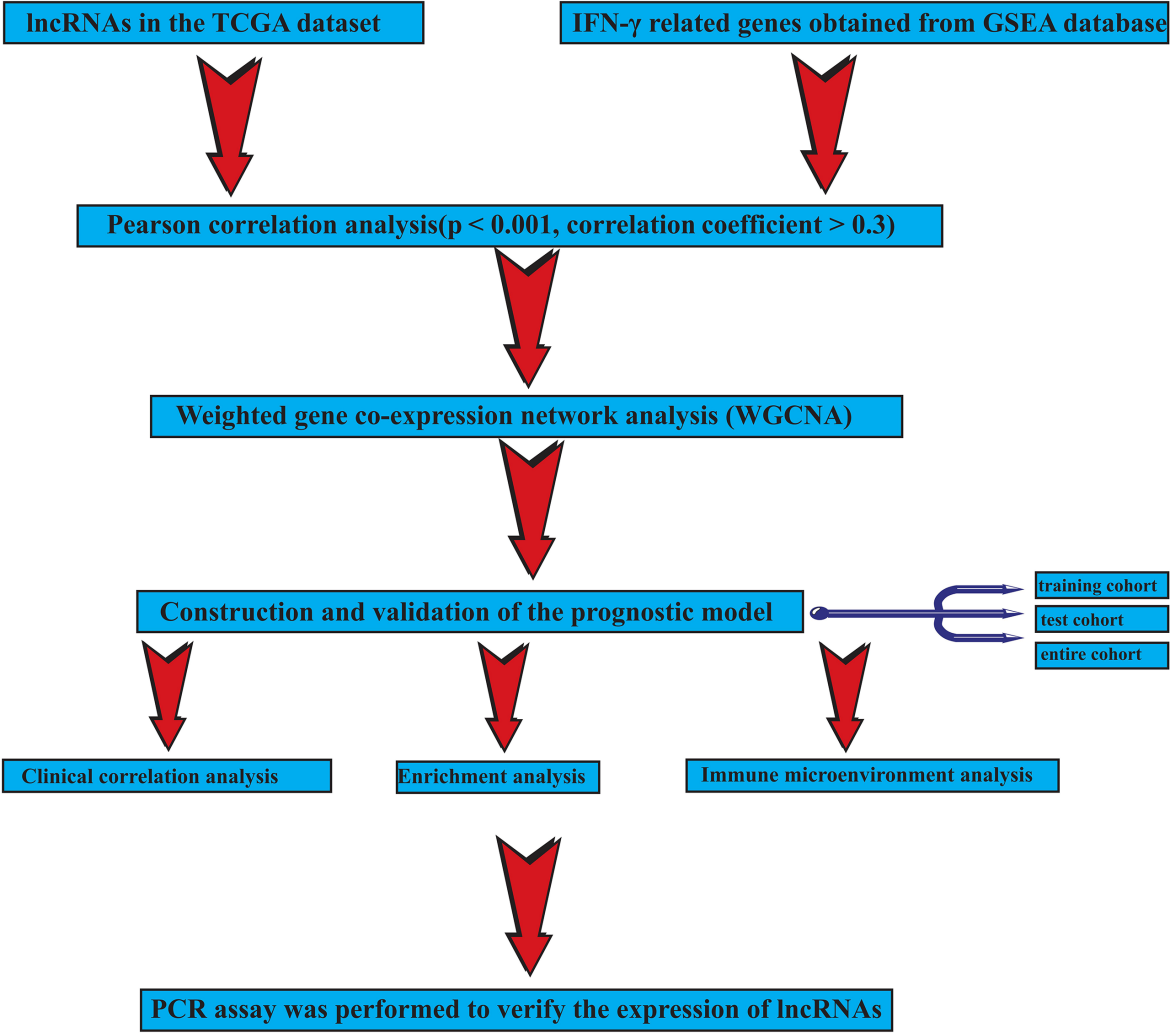
The flowchart.

Our workflow is shown in **Figure 1**.

### Identification of prognostic interferon gamma-related long non-coding RNAs in endometrial carcinoma

**Figure 2:**
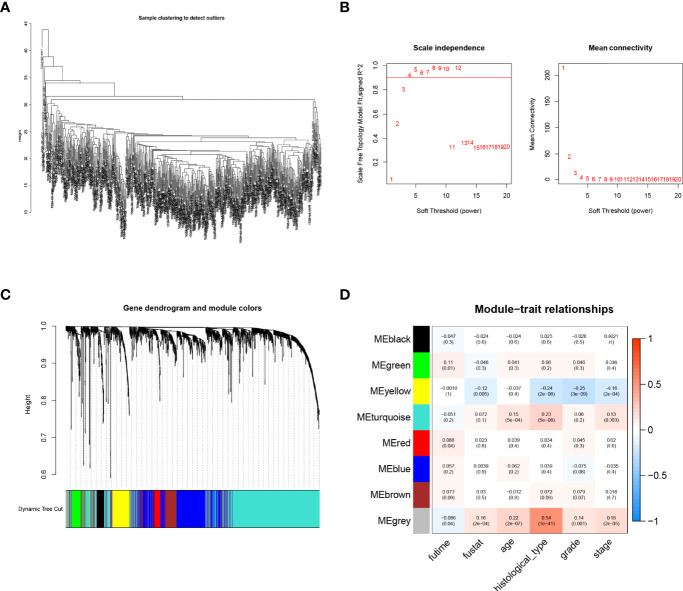
Weighted co-expression network analysis (WGCNA). **(A)** Sample clustering tree. **(B)** Soft threshold selection. As the threshold increased, the R^2^ value increased and crossed 0.8. When the number of modules is 8, the model is more stable. **(C, D)** Distribution and correlation of each module. Through correlation analysis between different modules and phenotype (survival time and survival status), it was finally found that green, red, and yellow modules were significantly positively correlated with endometrial carcinoma (EC)prognosis.

**Figure 3:**
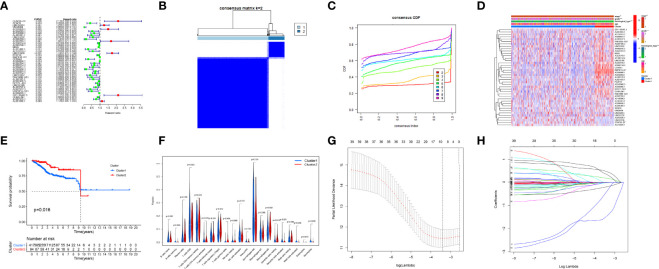
Consensus clustering analysis and the construction of the prognostic signature. **(A)** Univariate Cox regression. **(B, C)** Endometrial carcinoma (EC) patients of The Cancer Genome Atlas (TCGA) cohort can be classified into two clusters efficiently. **(D)** Relationship between clusters and clinical characteristics. **(E)** Cluster 2 had a more significant relationship with higher survival probability (*p*-value < 0.05). **(F)** The levels of immune cell infiltration between the two clusters were different. **(G, H)** Least absolute shrinkage and selection operator (LASSO) regression.

A total of 1,700 IFN-γ-related lncRNAs were obtained by co-expression analysis. Then weighted co-expression network analysis (WGCNA) was used to screen lncRNAs that were most correlated with clinical traits, i.e., survival time and survival status (**Figure 2A**). It was found that as the threshold increased, the R^2^ value increased and crossed 0.8. When the number of modules is 8, the model is more stable (**Figure 2B**). A total of 8 modules were identified from the co-expression network (**Figures 2C, D**). Among all non-gray modules, green, red, and yellow modules have the most significant correlation with survival time and survival status (**Figure 2D**). A total of 260 lncRNAs in the green, red, and yellow modules were selected for univariate Cox regression analysis, and 40 lncRNAs were obtained for subsequent cluster analysis (*p*-value < 0.05, **Figure 3A**).

### Based on the expression of interferon gamma-related long non-coding RNAs, a consensus clustering analysis was performed

Consensus clustering was conducted and indicated that patients in TCGA cohort can be classified into two clusters efficiently (**Figures 3B, C**). A sample correlation heatmap was created to depict the relationship between clusters and clinical characteristics (**Figure 3D**). KM survival curves demonstrated that cluster 2 had a more significant relationship with a higher survival probability (*p*-value < 0.05, **Figure 3E**). **Figure 3F** shows the differences in immune cell infiltration between the two clusters.

### Establishment and validation of a prediction model based on interferon gamma-related long non-coding RNAs

To avoid overfitting of IFN-γ-related lncRNAs, the LASSO algorithm was utilized to construct a prediction model (**Figures 3G, H**). We consequently obtained a precise formulation: CFAP58-DT * 0.4686 + LINC02014 * 0.1798 + UNQ6494 * 1.4725 + AC006369.1 * −0.4049 + NRAV * −0.0066 + BMPR1B-DT * −0.0004 + AC068134.2 * −0.0235 + AP002840.2 * −0.1923 + GS1-594A7.3 * 0.3475 + OLMALINC * −0.0112.

**Figure 4:**
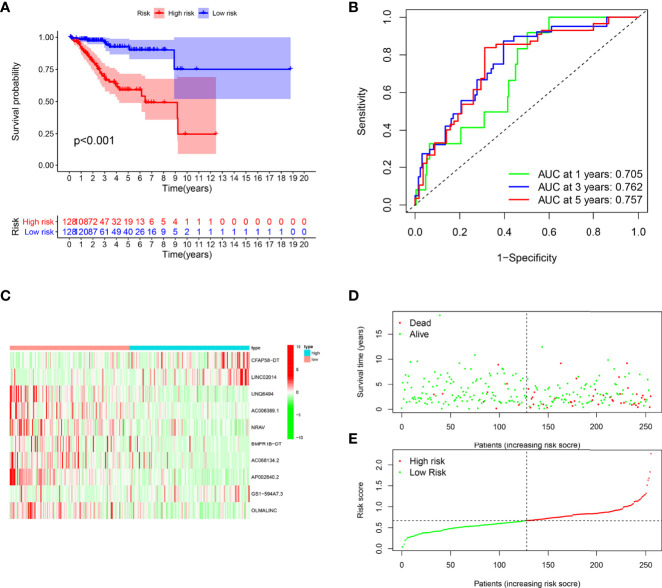
Evaluation of the prognostic value of this signature in training cohort. **(A)** Survival analysis showed a worse prognosis in the high-risk group (*p* < 0.001). **(B)** The 1-, 3-, and 5-year area under the curve (AUC) values of the signature are 0.705, 0.762, and 0.757, respectively. **(C)** Expression heatmaps of 10 lncRNAs in signature. **(D, E)** Survival status and risk score status of training cohort.

**Figure 5:**
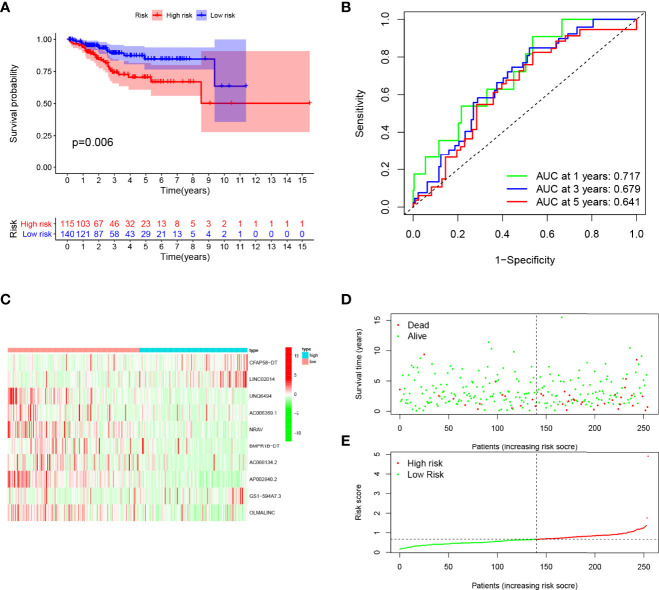
Evaluation of the prognostic value of this signature in validation cohort. **(A)** Survival analysis showed a worse prognosis in the high-risk group (*p* = 0.006). **(B)** The 1-, 3-, and 5-year area under the curve (AUC) values of the signature are 0.717, 0.679, and 0.641, respectively. **(C)** Expression heatmaps of 10 lncRNAs in signature. **(D, E)** Survival status and risk score status of validation cohort.

**Figure 6:**
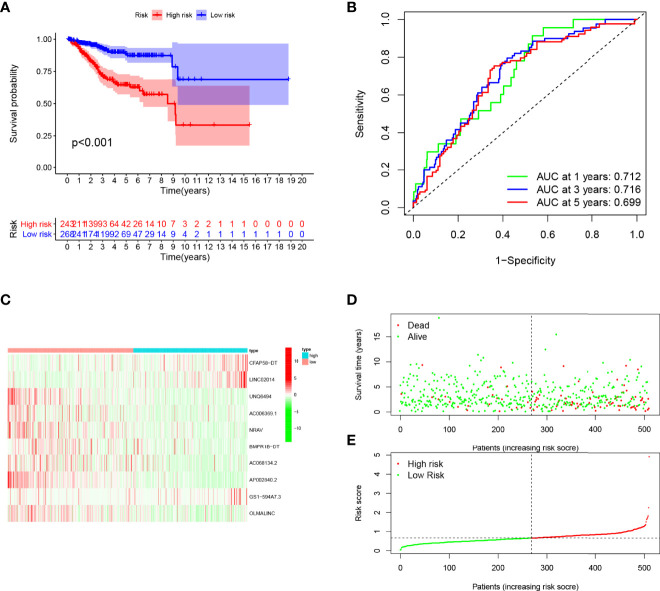
Evaluation of the prognostic value of this signature in entire cohort. **(A)** Survival analysis showed a worse prognosis in the high-risk group (*p* < 0.001). **(B)** The 1, 3, and 5-year area under the curve (AUC) values of the signature are 0.712, 0.716, and 0.699, respectively. **(C)** Expression heatmaps of 10 lncRNAs in signature. **(D, E)** Survival status and risk score status of entire cohort.

Patients in the training cohort were divided into the high- and low-risk groups based on their median risk score, with high-risk patients having a higher proportion of death occurrences (**Figure 4**). Patients in the high-risk group had lower survival outcomes than those in the low-risk group, according to the Kaplan–Meier analysis (**Figure 4A**). The area under the curve (AUC) for 1-year, 3-year, and 5-year OS was 0.717, 0.697, and 0.641, respectively, according to ROC curve analysis (**Figure 4B**). **Figures 3C–E** show the survival status of patients and the signature risk score in the low- and high-risk categories. The heatmap clearly illustrated the relationship between the two risk groups and part of the clinical information (**Figure 4C**). Also, the expression of the prognosis-related risk lncRNAs was demonstrated plainly. CFAP58-DT and LINC02014 were highly expressed in the high-risk group, while UNQ6494, AC006369.1, NRAV, AP002840.2, and OLMALINC were highly expressed in the low-risk group. Similar results observed in the test and entire cohort for validation are shown in **Figures 5**, **6**, indicating a great diagnostic capability of the prediction model.

### Correlation between clinical parameters and prediction model

**Figure 7:**
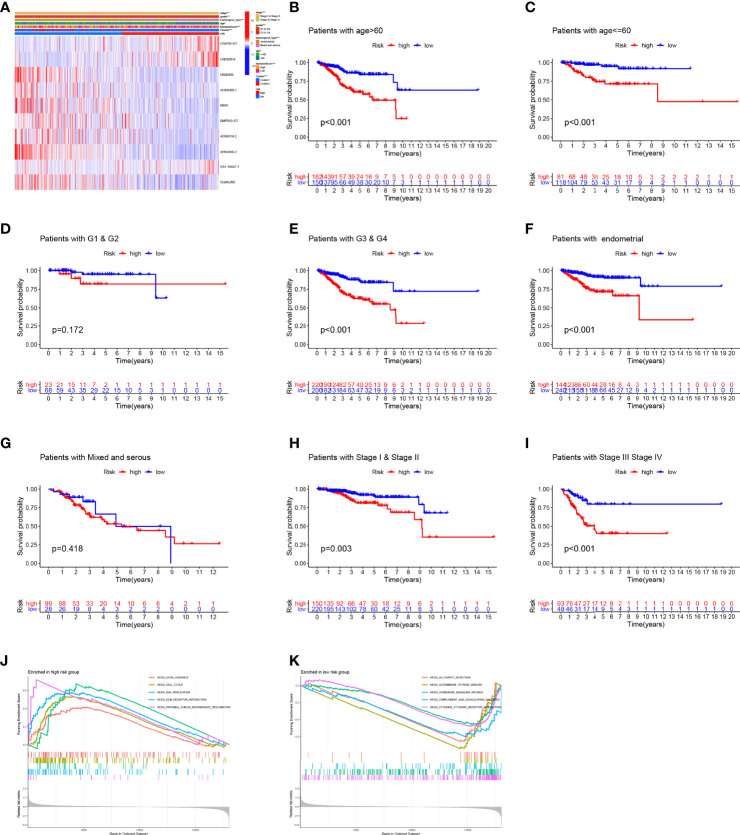
Correlation between clinical parameters and prediction signature. **(A)** The detailed characteristic distribution of two groups. **(B–I)** The prognostic model could effectively discriminate the prognosis of the patients stratified by different clinical factors (age, grade, histological type, and stage). **(J, K)** GSEA analysis.

To explore the relationship between clinical parameters and the prediction model, the detailed characteristic distribution of the two groups is shown in **Figure 7A**. Specifically, the risk score was significantly different in patients stratified by the clinical factors, including age, grade, histological type, stage, and cluster (**Supplementary Figure 1**). The Kaplan–Meier survival analysis according to the prognostic signature stratified by clinicopathological factors was further conducted, and the prognostic model could effectively discriminate the prognosis of the patients stratified by different clinical factors (age, grade, histological type, and stage, **Figures 7B–I**). Accordingly, patients included in the high-risk group have a poor prognosis regardless if they are over 60 or not. Aside from this, the high-risk group showed poor prognostic condition in stage, grade, and pathology subgroups, which indicated the efficiency of the established model in distinguishing the prognosis of EC patients.

### Pathway enrichment analysis of genes associated with the high- and low-risk groups

Genes associated with high risk are enriched in axon guidance, cell cycle, DNA replication, extracellular matrix (ECM)–receptor interaction, and proximal tubule bicarbonate reclamation (**Figure 7J**), whereas genes associated with low risk are enriched in allograft rejection, autoimmune thyroid disease, chemokine signaling pathway, complement and coagulation cascades, and cytokine–cytokine receptor interaction (**Figure 7K**).

### Immune context of prediction model

**Figure 8:**
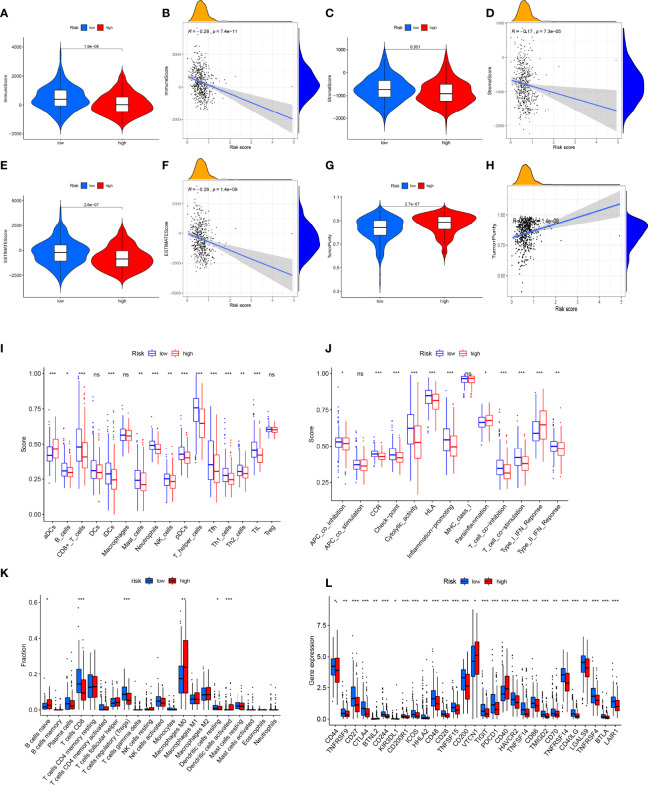
Analysis of immune microenvironment. **(A, B)** Immune score was lower in the high-risk group, and there was a significant negative correlation with risk scores. **(C, D)** Stromal score was lower in high-risk group, and there was a significant negative correlation with risk score. The **(E, F)** ESTIMATE score was lower in the high-risk group and significantly negatively correlated with the risk score. **(G, H)** Tumor purity was higher in the high-risk group, and there was a significant positive correlation with risk score. **(I–K)** Analysis of infiltration level of immune cells. **(L)** Expression analysis of immune checkpoint-related genes. *P<0.05, **P<0.01, ***P<0.001, ns, no significance.

It is known that immune cell infiltration affects the survival and tumor metastasis of patients. A violin plot of the immune microenvironment differences demonstrated that the high-risk group had a trend to gain low immune score, stromal score, and estimate score and eventually develop a high-purity tumor (**Figures 8A–H**). Through the CIBERSORT algorithm and ssGSEA algorithm, the low-risk group showed a higher proportion of immune cells than the high-risk group (**Figures 8I–K**), which indicates that patients in the high-risk group had a relatively low immune status. We also checked the expression changes of immune checkpoints, which might be indicative of the clinical response of immunotherapies, and we found the patients of the low-risk group had a higher expression of immune checkpoints as compared with the high-risk group (**Figure 8**). These findings may explain the different overall survival of the two groups. Based on the observation, we performed targeted drug tests on experimental animals and recorded the curative effect of the 16 kinds of drugs (**Supplementary Figure 2**). TNFRSF9, CD27, CTLA4, BTNL2, CD244, CD200R1, ICOS, HHLA2, CD48, CD28, TNFSF15, CD200, TIGIT, PDCD1, CD40, HAVCR2, TNFSF14, CD86, TMIGD2, CD70, TNFRSF14, CD40LG, LGALS9, TNFRSF4, BTLA, and LAIR1 were expressed significantly differently in the high- and low-risk groups.

Additionally, we screened the expression of *N* ^6^-methyladenosine RNA methylation and stem cell pathway-related genes in the two groups (**Supplementary Figures 2B–D**). As a result, YTHDF1, YTHDC2, RBM15, and WTAP were observed to have high expression in the high-risk group, which reflected the poor prognosis indirectly.

### Independent prognostic analysis and construction of a nomogram based on the established model

**Figure 9:**
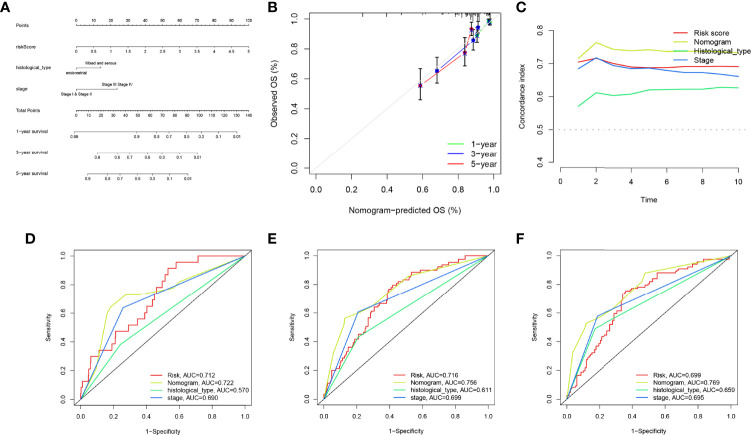
Construction and evaluation of the nomogram. **(A)** The nomogram was developed for clinical application. **(B)** Calibration curves of the nomogram we developed. **(C)** The nomogram achieved a significantly higher c-index value than other clinical factors, meaning a better predictive accuracy for endometrial carcinoma (EC). **(D–F)** The area under the curve (AUC) values of the nomogram for 1-, 3-, and 5-year overall survival (OS) were higher than 0.7 and other clinical factors, indicating that the nomogram was reliable.

Univariate and multivariate Cox regression analyses indicated that the risk score can serve as an independent prognostic factor in EC (**Table 1**). Further, tests and entire cohorts were enrolled, and the same results were observed (**Tables 2** and **3**). Based on the results of independent prognostic analysis, a nomogram was developed for clinical application (**Figure 9A**). The calibration curves of the nomogram we developed showed a great consistency between the actual observation and the nomogram prediction (**Figure 9B**). As is shown in **Figure 9C**, the nomogram achieved a significantly higher c-index value than other clinical factors, meaning a better predictive accuracy for EC.

**Table 1:** Independent prognostic analysis of training cohort.

	Univariate analysis				Multivariate analysis				
	HR	HR.95L	HR.95H	*p*-Value		HR	HR.95L	HR.95H	*p*-Value	
**age**	2.310787	1.174746	4.54544	0.015244		2.101931	0.997104	4.430943	0.050898	
**histological type**	2.723841	1.540402	4.816476	0.00057		0.995563	0.507416	1.953321	0.989683	
**grade**	4.132983	1.281972	13.32444	0.017508		1.686432	0.477843	5.951856	0.416654	
**stage**	3.370365	1.901033	5.975361	3.20E−05		2.829301	1.467133	5.456181	0.00191	
**risk Score**	6.640916	3.541853	12.45161	3.57E−09		4.685257	2.19568	9.997646	6.50E−05	

**Table 2:** Independent prognostic analysis of test cohort.

	Univariate analysis				Multivariate analysis			
	HR	HR.95L	HR.95H	*p*-Value	HR	HR.95L	HR.95H	*p*-Value
**age**	1.358867	0.703253	2.625685	0.361529	NA	NA	NA	NA
**histological type**	3.430404	1.847279	6.370272	9.49E−05	2.457144	1.307522	4.617558	0.005222
**grade**	2.714988	0.826275	8.920951	0.099854	NA	NA	NA	NA
**stage**	5.346375	2.818812	10.14035	2.85E−07	4.087041	2.108869	7.920787	3.04E−05
**risk Score**	2.56945	1.692921	3.899812	9.29E−06	1.997949	1.201528	3.322268	0.00764

**Table 3:** Independent prognostic analysis of entire TCGA cohort.

		Univariate analysis			Multivariate analysis			
	HR	HR.95L	HR.95H	*p*-Value	HR	HR.95L	HR.95H	*p*-Value
**age**	1.778212	1.112123	2.843245	0.016228	1.566681	0.956845	2.56519	0.074324
**histological type**	3.043526	2.003172	4.62419	1.84E−07	1.643683	1.039595	2.598794	0.033495
**grade**	3.363109	1.467057	7.709655	0.004165	1.342176	0.551863	3.264285	0.516335
**stage**	4.116248	2.699981	6.275414	4.82E−11	3.043751	1.918006	4.830235	2.31E−06
**risk Score**	3.106371	2.316925	4.164804	3.55E−14	2.532946	1.754055	3.657704	7.15E−07

TCGA, The Cancer Genome Atlas.

Moreover, the AUC values of the nomogram for 1-, 3-, and 5-year OS were higher than 0.7 and other clinical factors, indicating that the nomogram was reliable (**Figures 9D–F**).

### PCR was used to verify the expression of long non-coding RNAs in the model in endometrial carcinoma

To further verify our analysis results, PCR experiments were carried out. The results showed that BMPR1B-DT and UNQ6494 were significantly upregulated in endometrial carcinoma (**Supplementary Figure 3**; *p* < 0.05). However, LINC02014 and NRAV were significantly downregulated in endometrial carcinoma (**Supplementary Figure 3**; *p* < 0.05). PCR showed no statistical difference in the expression of other lncRNAs.

## Discussion

Endometrial cancer is one of the most common malignant tumors of the female reproductive tract, mainly in postmenopausal women, but a number of young women are still affected (31). Patients with early, localized EC who have undergone surgical treatment have a good prognosis, with a 5-year survival rate of more than 80% (32). However, patients with advanced EC often have lymph node or distant metastasis, poor prognosis, and limited treatment (33). Therefore, it is necessary to explore new biomarkers for prognostic stratification of EC patients and to provide a reference for precise treatment. IFN-γ, one of the most common types of immune cytokines, has been preliminarily elucidated to play a key role in the tumor immune microenvironment (34). However, the prognostic value and mechanisms of IFN-γ-related genes and lncRNAs in EC remain unclear. In-depth exploration is necessary to uncover the role of the IFN-γ pathway in EC. Moreover, endometrial carcinoma is a group of tumors with heterogeneity, including pathogenesis, growth characteristics, treatment response, and prognosis (35). Among them, identifying the prognostic difference in endometrial cancer can provide a reference for early intervention and precise treatment (36). Previously, the International Federation of Gynecology and Obstetrics (FIGO) grading based on the degree of differentiation of glands has been widely used in clinical diagnosis and treatment of endometrial cancer and prognosis assessment (37). However, in genomics advances, genomic-based endometrial cancer typing is becoming more and more attractive (38). Moreover, the identification of genomic instability or microsatellite instability subtypes can provide a reference for immunotherapy (39). Our study establishes the prognostic signature of IFN-γ-related lncRNAs for the first time, in which the grouping of endometrial cancer patients not only can guide prognostic assessment but also can help in understanding the differences in the immune microenvironment.

In this study, we analyzed endometrial cancer data from TCGA database using a variety of bioinformatics methods. First, the interferon gamma-related lncRNAs were divided into a total of eight modules by WGCNA, among which the gray module was most correlated with the interferon gamma phenotype of EC. Subsequently, lncRNAs in the gray module were extracted for further analysis. Consensus clustering analysis found that these lncRNAs could well divide EC patients in TCGA database into two clusters, with significant prognostic differences between the two clusters. Cox regression and LASSO regression were used to construct prognostic signatures. Each patient could be calculated with a risk score = CFAP58-DT * 0.4686 + LINC02014 * 0.1798 + UNQ6494 * 1.4725 + AC006369.1 * −0.4049 + NRAV * −0.0066 + BMPR1B-DT * −0.0004 + AC068134.2 * −0.0235 + AP002840.2 * −0.1923 + GS1-594A7.3 * 0.3475 + OLMALINC * −0.0112. This allowed the value of risk to be assessed for each patient, allowing patients from various cohorts to be separated into the high-risk and low-risk groups, with the high-risk group having a worse prognosis. Varying levels of immune cell infiltration, medication sensitivity, and immunological checkpoint levels were also associated with different risk scores.

The signature we constructed consists of 10 lncRNAs, and many studies have preliminarily explained the role of these 10 lncRNAs in cancer. Sui et al. found that UNQ6494 was a poor prognostic marker for lung adenocarcinoma (40). The role of NRAV in cancer has been repeatedly confirmed. Wang et al. found that NRAV can mediate the activation of the Wnt/β-catenin signaling pathway to promote the proliferation and invasion of hepatocellular carcinoma (41). Lin et al. discovered that BMPR1B-DT is a prognostic marker of ovarian cancer and is associated with drug sensitivity (42). The role of OLMALINC in osteosarcoma was confirmed by He et al., who found that it was highly correlated with the immune microenvironment of osteosarcoma and could assess patient outcomes (43). Our study is the first to reveal the role of the aforementioned lncRNAs in endometrial cancer and their value in the tumor microenvironment.

The development of cancer is associated with the activation of multiple pathways, and the exploration of these key pathways will help to identify vulnerable points of cancer and enable more effective treatment of cancer (44). Our study found that the high-risk group was associated with enrichment of cell cycle, DNA replication, and ECM–receptor interaction pathways, which are highly related to cancer cell replication and growth, which may be one of the reasons for poorer prognosis in the high-risk group. Cancer cells are known to be in a highly active state of metabolism and replication (45). In the process of unlimited replication, cancer cells accumulate a large number of mutations and instabilities, which may be potential therapeutic targets for cancer (46). Through enrichment analysis, we found that the high-risk group was associated with significant enrichment of DNA replication and cell cycle pathways, which may be potentially related to IFN-γ, providing a reference for us to understand its complex crosstalk. The role of ECM-related signaling pathways in cancer is central and variable (47). Dysregulation of ECM in cancer results in adherent junctions, loss of tissue polarity, and epithelial–mesenchymal transition (EMT) (48). Moreover, dysregulation of ECM is also associated with the chronic inflammatory background of cancer, where oversecretion of cytokines stimulates downstream signaling pathways, promotes tumor growth, and mediates the development of drug resistance (49). The activation of the ECM signaling pathway in the high-risk group may be the underlying mechanism of IFN-γ in regulating endometrial cancer growth, which provides a reference for guiding related treatment.

Currently, several IFN-γ-related signatures have been built. Yan et al. constructed the prognostic signature associated with IFN-γ-activated CD8+ T cells to assess the prognosis and immune response level of melanoma patients using a weighted co-expression network analysis (50). Yao et al. constructed the signature of seven IFN-γ-related lncRNAs to assess prognosis in patients with lung adenocarcinoma, in which the high-risk group had a worse prognosis and was associated with high levels of tumor-promoting immune cells (51). Liu et al. constructed an IFN-γ-related signature in renal clear cell carcinoma, where the high risk score is associated with immunosuppressive microenvironment and drug resistance (52). In addition, there are many new signatures being built in multiple tumors. Liu et al. constructed the signature of mutation-derived genome instability-associated lncRNAs, in which risk score was negatively correlated with prognosis and immune cell infiltration (53). Yuan et al. explored the role of M5C-associated lncRNA in pancreatic ductal adenocarcinoma and found that the M5C-associated signature is a prognostic marker and immune evaluation indicator of pancreatic ductal adenocarcinoma (54). Through in-depth analysis, Gao et al. found that EMT-related lncRNAs constitute a prognostic signature in pancreatic cancer, with a significantly worse prognosis in the high-risk group (55). Liu et al. also constructed an immune-related lncRNA signature in endometrial cancer through bioinformatics analysis, and different risk scores were associated with different prognoses and immune statuses (56). In contrast to these published studies, our study constructed the prognostic signature of IFN-γ-related lncRNAs for the first time to assess the prognosis and immune microenvironment of endometrial cancer. Our signature has good robustness in evaluating the prognosis of EC, and it can be seen that the prognosis of patients in the high-risk group is significantly worse in the training cohort, validation cohort, and entire cohort. Second, the ROC curve shows that our signature has a high AUC value and good accuracy. Our signature also reveals the immune landscape and differences in drug sensitivity between different risk groups to provide a reference for their treatment.

## Conclusion

Our study provides an in-depth analysis of the role of IFN-γ-related lncRNAs in endometrial cancer. The prognostic signature constructed by us can effectively evaluate the prognosis and immune status of patients with endometrial cancer. However, there are limitations to our study, as we lack *in vivo* and *in vitro* experiments to confirm our conclusions. Meanwhile, we lack the validation of cohort from our center, and we will improve it in the future. More studies are expected to explore the significance of IFN-γ in endometrial cancer.

## Data availability statement

The datasets presented in this study can be found in online repositories. The names of the repository/repositories and accession number(s) can be found in the article/**supplementary material**.

## Ethics statement

This study was reviewed and approved by Ethics Committee of Nantong Haimen People’s Hospital. The patients/participants provided their written informed consent to participate in this study.

## Author contributions

CG designed the study. CG and ZZ were involved in database search and statistical analyses. CG, ZZ, CL, and LH were involved in the writing of the manuscript and its critical revision. CG, XZ, and SH were responsible for the submission of the final version of the paper. All authors approved the final version. All authors agree to be accountable for all aspects of the work.

## Funding

This research was supported by the National Natural Science Foundation of China (82173554).

## Acknowledgments

We are very grateful for the data provided by databases such as TCGA and GEO.

## Conflict of interest

The authors declare that the research was conducted in the absence of any commercial or financial relationships that could be construed as a potential conflict of interest.

## Publisher’s note

All claims expressed in this article are solely those of the authors and do not necessarily represent those of their affiliated organizations, or those of the publisher, the editors and the reviewers. Any product that may be evaluated in this article, or claim that may be made by its manufacturer, is not guaranteed or endorsed by the publisher.

## Abbreviations

EC, endometrial carcinoma; TCGA, The Cancer Genome Atlas; GSEA, gene set enrichment analysis; WGCNA, weighted gene co-expression network analysis; LASSO, least absolute shrinkage and selection operator; ECM, extracellular matrix; EMT, epithelial–mesenchymal transition.
