# Supplementary figures and images for: The IFN-γ-related long non-coding RNA signature predicts prognosis and indicates immune microenvironment infiltration in uterine corpus endometrial carcinoma
Source: Front Oncol. 2022 Jul 26;12:955979. doi: 10.3389/fonc.2022.955979 (PMC9360323; doi:10.3389/fonc.2022.955979)

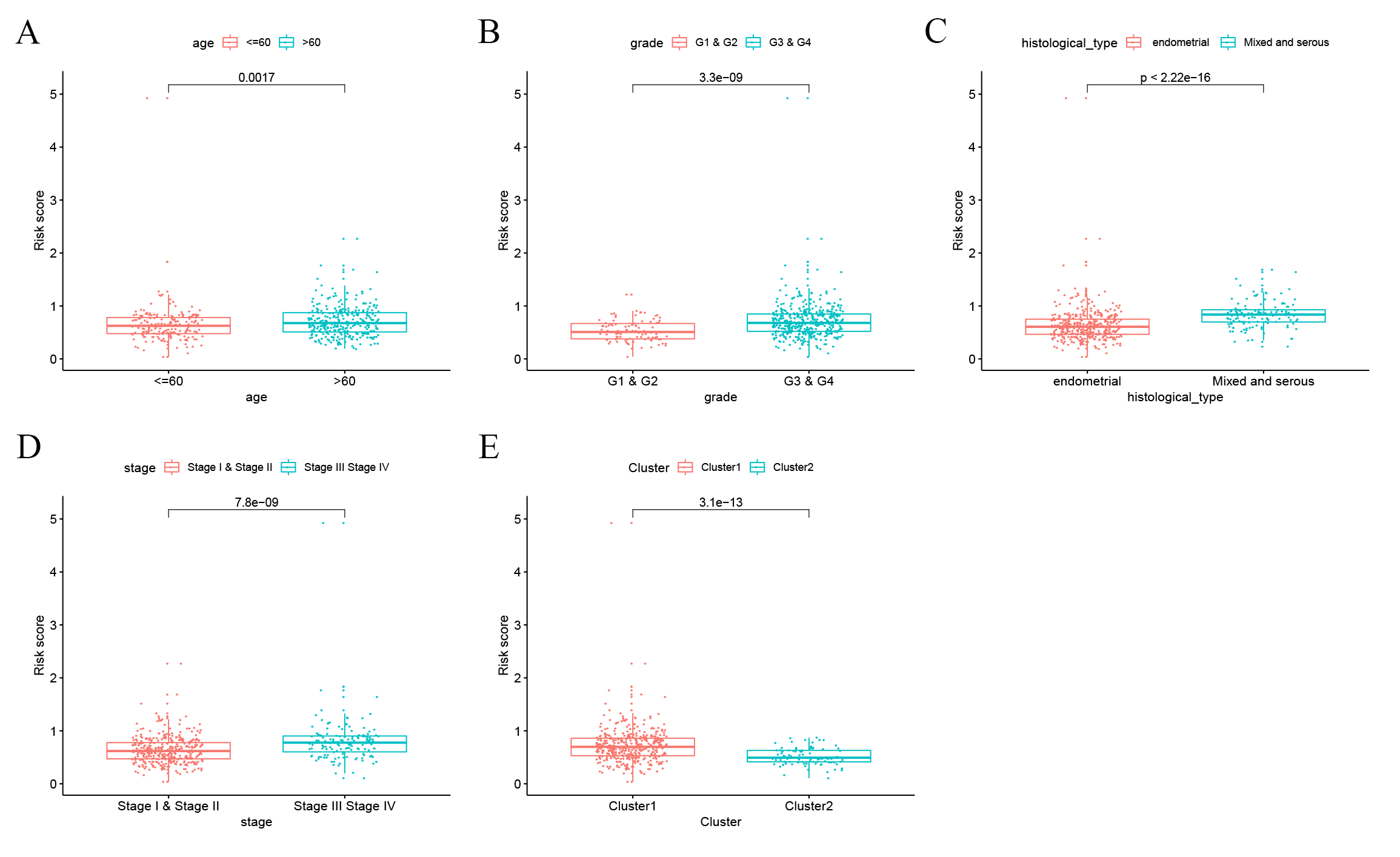

Supplement: Supplementary file 1 [file Image_1.tif]

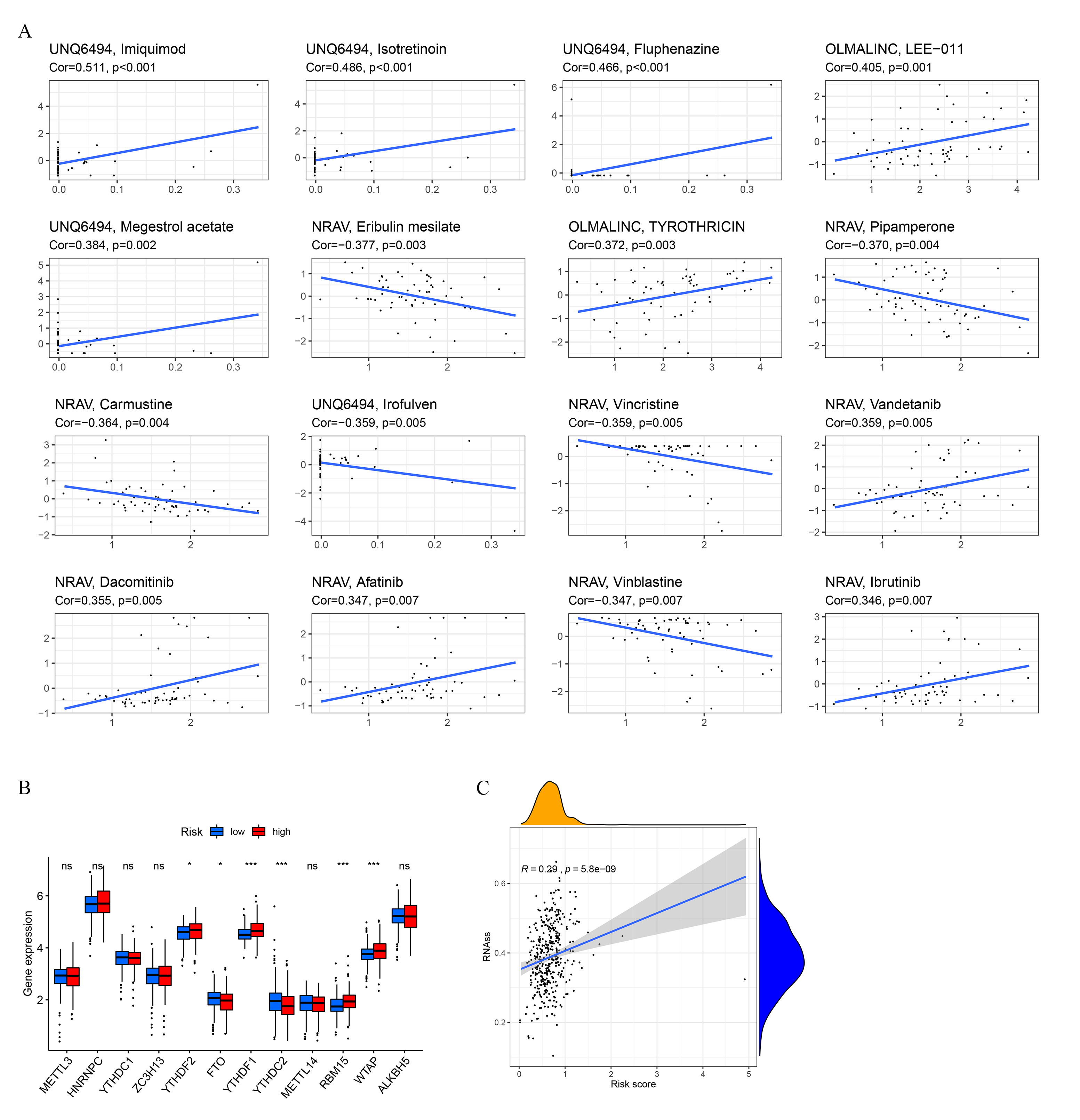

Supplement: Supplementary file 2 [file Image_2.tif]

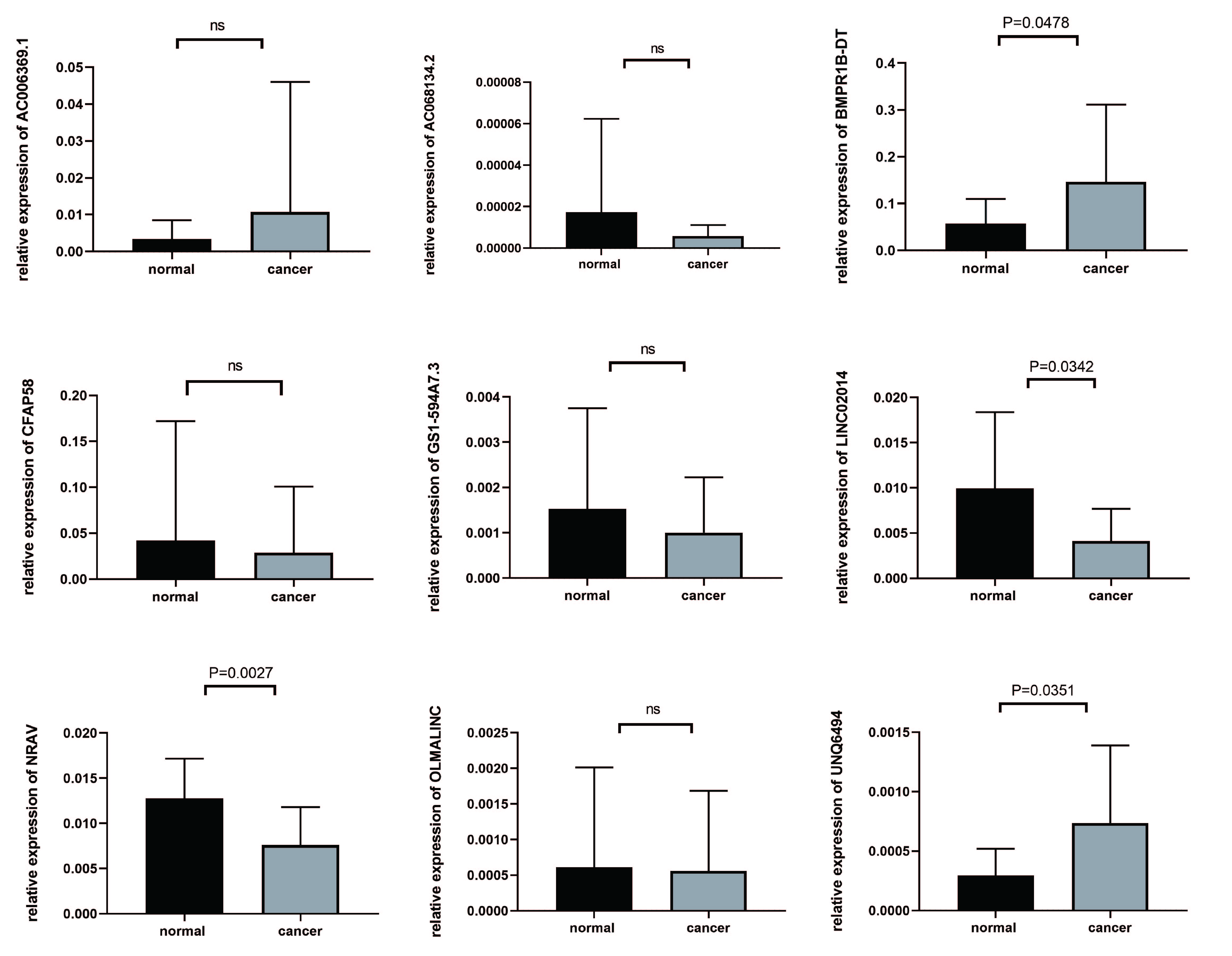

Supplement: Supplementary file 3 [file Image_3.jpeg]
